# Health-related quality of life and work productivity in UK patients with HER2-positive breast cancer: a cross-sectional study evaluating the relationships between disease and treatment stage

**DOI:** 10.1186/s12955-020-01603-w

**Published:** 2020-11-02

**Authors:** Mark Verrill, Andrew M. Wardley, Jenny Retzler, Adam B. Smith, Catherine Bottomley, Sorcha Ní Dhochartaigh, Irwin Tran, Iain Leslie, Peter Schmid

**Affiliations:** 1 The Newcastle Upon Tyne Hospitals NHS Foundation Trust, Newcastle, UK; 2 Division of Cancer Sciences, University of Manchester and the Christie NHS Foundation Trust, Manchester, UK; 3 York Health Economics Consortium, York, UK; 4 Department of Psychology, University of Huddersfield, Huddersfield, UK; 5 pH Associates Ltd (an Open Health Company), Marlow, UK; 6 Roche Products Ltd, Welwyn Garden City, UK; 7 Queen Mary University of London, London, UK

**Keywords:** Breast cancer, Work productivity, Heath-related quality of life, Early disease, Metastatic disease

## Abstract

**Background:**

The impact of different disease stages and treatment for human epidermal growth factor 2 positive (HER2-positive) breast cancer (BC) on work productivity and health-related quality of life (HRQoL) is poorly understood.

**Methods:**

This was a UK cross-sectional study of 299 adult patients with HER2-positive early or metastatic BC (NCT03099200). Productivity was assessed using the work productivity and activity impairment scale; HRQoL was measured using EuroQol-5 Dimensions-5 levels (EQ-5D-5L), and Functional Assessment of Cancer Therapy Breast (FACT-G and -B) instruments. Three balanced patient groups were recruited: (1) early BC on treatment post-surgery, (2) early BC after completion of adjuvant treatment, (3) during metastatic BC treatment. Between-group comparisons were performed using an analysis of variance.

**Results:**

Group 1 comprised 89 patients, Group 2, 108 and Group 3, 102. Age, ethnicity and comorbidities were similar across groups. Patients in Group 3 reported more often being unable to work (significant Bonferroni adjusted *p* < 0.003). Proportions of employed patients were 50.6%, 50.9% and 27.5% in Groups 1, 2 and 3, respectively. For patients in part-time employment, the number of hours worked was significantly higher in Group 2 patients versus Group 3 (*p* = 0.002). Group 2 also had significantly lower levels of work absenteeism and overall work impairment compared with Group 1 (*p* < 0.001). Patients in Group 3 reported worse health utility scores (*p* ≤ 0.002), moderate or worse problems in the EQ-5D-5L self-care and usual activity domains (*p* ≤ 0.001), and lower HRQoL as assessed by FACT summary scores (*p* < 0.001 for FACT-B and -G) than Groups 1 and 2. Poorer HRQoL was significantly associated with higher work impairment (*p* < 0.001), with the strongest relationships being observed between activity impairment and HRQoL (Pearson’s r: 0.67).

**Conclusions:**

Metastatic disease and treatment of HER2-positive BC adversely impacted on work productivity and HRQoL. The results of this study support the idea that being able to delay or prevent the metastatic recurrence of BC, for example by extending the time patients are in remission or at early stage of BC, has wider benefits in terms of patient productivity and HRQoL.

## Introduction

Breast cancer (BC) is the most common form of cancer in the UK, with over 50,000 new cases diagnosed each year [1]. Mortality rates for patients with BC have fallen by approximately 40% in the UK since the 1980s, and almost 80% of women diagnosed in England and Wales now survive the disease for 10 years or more owing to advances in screening, diagnosis and treatment [2].

Human epidermal growth factor 2 (HER2) is a protein that stimulates normal breast cell division and growth. However, in 20–25% of BCs, known as HER2-positive BCs, the cancer cells express greater numbers of HER2 receptors [3] and HER2-positive breast cancers tend to grow more quickly and are more likely to recur and metastasise than HER2-negative breast cancers [3]. Furthermore, there is a significant association between HER2 over-expression and poor prognosis, with decreased disease-free survival and overall survival in node-positive patients [4].

Treatment of early BC typically aims to cure, with combinations of surgery and chemotherapy, radiotherapy, and targeted or hormonal therapy where indicated. Following completion of treatment, patients are considered to be in ‘remission’ and begin to resume normal life, with around 70% remaining free of their disease after 10 years [5]. In contrast, treatment of metastatic BC aims to extend life, while maintaining health-related quality of life (HRQoL), typically comprising sequential lines of chemotherapy, which for HER2-positive patients is combined with ongoing HER2-targeted therapy [6].

Improvements that lead to earlier diagnosis or more effective treatment are likely to increase the proportion of patients achieving and maintaining remission, whilst decreasing the proportion of patients with metastatic BC. This will intuitively result in direct patient benefits in terms of better HRQoL and ability to perform work and non-work related activities, with indirect economic benefits for society.

The clinical progression of patients with HER2-positive BC diagnosed with early or metastatic disease has been well characterised in clinical trials and real-world studies [7, 8]. In recent years significant progress has been made in the treatment of this aggressive subtype of BC, specifically with the approval of HER2-targetted agents in the both the early and metastatic settings. These treatment advances have impacted the natural history of the disease and added years to a patient’s prognosis in both the early and metastatic settings [9–11]. However, evidence on the broader implications of the different stages of BC treatment specifically in patients who are HER2-positive, including on patients’ ability to work, their usual activities and HRQoL in general, remains limited for the UK. Published estimates of work productivity in patients with BC have been heterogeneous across studies. A study in US patients with metastatic BC observed a 20–40% reported decrease in work productivity [2]; whilst in a study in Swedish and Dutch patients with early or advanced BC, work productivity reductions were approximately 70% for patients on treatment and 40% for patients who had completed treatment [12]. Studies have also shown a poorer rating on several dimensions of HRQoL, which remain in some patients 5–10 years after diagnosis [13, 14]; although some aspects such as emotional wellbeing and depression were not affected in most patients [13], even in those at advanced stage of BC and currently on treatment [14].

This real-world study (clinicaltrials.gov identifier: NCT03099200) aimed to assess how living in each stage of HER2-positive BC treatment (patients with early BC currently receiving adjuvant treatment; patients with early BC who have completed adjuvant parenteral therapy; and patients with metastatic BC) impacts directly on patients’ HRQoL and productivity, and indirectly on society in terms of cost of lost productivity, to help quantify the wider benefit of developing new interventions which delay or prevent the metastatic recurrence of BC.

## Methods

### Sample recruitment

This was a cross-sectional, observational study conducted between December 2016 and March 2017. Patients were recruited to attain relatively balanced groups in terms of sample size (planned sample size for each group n = 100) who were each representative of the general population of UK patients with BC in terms of treatment at the relevant stage. Group 1 comprised patients currently undergoing treatment for early BC (patients were selected to ensure adequate representation of patients treated with chemotherapy and targeted HER2 therapy [planned n = 40/100] and those treated with targeted HER2 therapy alone); Group 2 comprised patients with early BC who had completed treatment and were in remission (i.e. no longer receiving loco-regional treatment, chemotherapy or targeted HER2 therapy; patients may still have been receiving hormone therapy); Group 3 comprised patients receiving treatment for metastatic BC (patients were selected to ensure adequate representation of patients receiving first-line treatment [planned n = 50/100] and those receiving second/subsequent lines of treatment for metastatic BC). Two-hundred and ninety-nine patients with HER2-positive BC were enrolled into the study through physician referral using a mixed approach to recruitment (e.g. invited to participate at routine clinic appointments, telephone appointments or via a letter from their physician in order to reach those patients who did not require frequent clinic appointments) from 14 secondary and tertiary care centres across England, UK. Patients were eligible for enrolment at each site if they were aged ≥ 18 years at study start and had been diagnosed with early stage (stage I–III) or metastatic (stage IV) HER2-positive BC (confirmed by the study site physician). HER2-positive was defined as immunohistochemistry-positive and/or in situ hybridisation ≥ 2.0. Patients were excluded if they were unwilling or unable to consent, unable to complete HRQoL questionnaires, or had an Eastern Cooperative Oncology Group Performance Status ≥ 3 [15].

### Data collection

Patients enrolled into the study completed questionnaires, either at the clinic or at home, on socio-demographic characteristics, including education level and work status, as well as several surveys. Surveys completed included the work productivity and activity impairment (WPAI) survey, the EuroQol-5 Dimensions-5 levels (EQ-5D-5L) questionnaire (dimensions: mobility, self-care, usual activities, pain/discomfort, anxiety/depression; visual analogue scale [VAS] score; index values), and the Functional Assessment of Cancer Therapy-Breast (FACT-B) questionnaire (subscale scores: physical wellbeing, functional wellbeing, social wellbeing, emotional wellbeing, BC-specific symptoms; FACT-B total score; FACT-General score; Trial Outcome Index). Clinical staff collected data from participating patients’ medical records related to demographics, medical history, disease and treatment history, and current treatments.

The study was performed in accordance with the Declaration of Helsinki and UK ethical approval from the Health Research Authority Research Ethics Committee (approval 16/EE/0429) was in place prior to study commencement.

### Analysis

#### Data processing

WPAI subscales, EQ-5D-5L utility index, and FACT-B subscales were derived from patients’ data using published scoring algorithms [16–18]. The WPAI raw scores were converted into the four domains: absenteeism, presenteeism, work productivity loss and activity impairment.

The utility England tariff [16] reflects health states preferences in the UK population and was used as value set for the estimation of index values for the EQ-5D-5L. In addition, estimates using the utility UK crosswalk tariff [19] that associates values with the EQ-5D-3L equivalent profiles, were also estimated to enable potential comparisons of health utility with studies using the EQ-5D-3L. For domain scores of the FACT-B where ≥ 50% of patients provided answers, missing values were imputed using the mean of that domain score. All other missing values were not imputed and the proportion of missing values was computed for each survey (WPAI, FACT-B, EQ-5D-5L) within each of the three groups.

#### Data analysis

Data were analysed using SPSS (version 24.0). Descriptive statistics were calculated for patients’ characteristics (socio-demographics, disease history, current treatment and treatment history) and questionnaire scores (WPAI, EQ-5D-5L and FACT-B) for the full sample and stratified by patient group. Inter-group differences were evaluated using analysis of variance (ANOVA) for continuous variables, ordinal regression for ordinal variables, and Chi-square (χ^2^) analysis for categorical variables. A *p* value < 0.05 was considered statistically significant. The lower bound of the established minimal important differences [20] was used to interpret the clinical relevance of differences on the FACT-B scales.

Where appropriate, post hoc analyses were conducted to interpret significant main effects: pairwise t-test for ANOVA, and analysis of adjusted residuals for χ^2^ tests. Multiplicity of tests was accounted for in each post hoc analysis by applying a Bonferroni correction (level of significance post hoc *p* < 0.0167 for WPAI, threshold for FACT-B for each post hoc test is provided in the results section). Where the assumption of homogeneity of variance for ANOVA test was violated, as tested by Levene test, the Brown–Forsythe F-ratio and Greenhouse–Geisser *p* values were estimated, and the Games–Howell post hoc tests were conducted instead. This was the case for WPAI domains of absenteeism, overall work impairment and activity impairment variables (but not for presenteeism); for EQ-5D-5L VAS and utility index values; FACT-B dimensions of physical wellbeing, social wellbeing, emotional wellbeing, functional wellbeing, and FACT-G score variables.

To assess how HRQoL was related to productivity and activity impairment, Pearson’s correlation coefficients were calculated for HRQoL summary scores (EQ-5D-5L VAS; FACT-B total and FACT-G total) and FACT-B domains in relation to WPAI subscale scores for all three groups of patients combined. These correlations were used to guide linear multiple regression models assessing whether specific domains of HRQoL predicted (1) absenteeism, and (2), presenteeism. Physical wellbeing, functional wellbeing and BC-specific symptoms were entered into the model predicting absenteeism using a forced entry technique. Bonferroni adjusted *p* values of *p* < 0.008 were used in these analyses as a threshold for significance.

## Results

A total of 299 eligible female patients from 14 sites were recruited to the study: 89 were assigned to Group 1 (patients currently undergoing targeted treatment for early BC); 108 were assigned to Group 2 (patients with early BC who had completed treatment and were in remission); and 102 were assigned to Group 3 (patients receiving treatment for metastatic BC). The proportion of missing data was < 6% for all the surveys conducted within each of the three groups. Recruitment into groups 1 and 3 was broadly as planned to mirror the proportion of patients in the UK HER2-positive BC population receiving types of treatment that could be expected to impact productivity and HRQoL (Group 1: with chemotherapy and HER2 targeted therapy [planned n = 40/100, actual n = 27/89]; Group 3: first-line treatment [planned n = 50/100, actual n = 55/102]; Additional file 1: Table S2).

### Patient demographics and clinical characteristics

Patient groups did not differ significantly with regard to age, level of education, presence of comorbidities or hormone receptor positivity at study enrolment (Table 1 and Additional file 1: Table S1). Mean age of patients was 55.0 (SD: 11.1) years, 57.7 (10.6) years and 55.3 (11.2) years, in Groups 1, 2 and 3, respectively. Median time since diagnosis of early BC was 9.0 months (interquartile range [IQR]: 6.0 months), 45.0 months (32.0) and 79.5 months (82.0) in Groups 1, 2 and 3, respectively; and the median time since diagnosis of metastatic BC in Group 3 was 30.0 (37.0) months (Table 1). In Group 3, over a quarter of patients (26.5%) had de novo metastatic cancer; over three-quarters (75.5%) had visceral metastases and approximately a quarter (24.5%) had central nervous system (CNS) metastases. The treatment status of patients at study enrolment is summarised in Additional file 1: Table S2. Group 2 patients had completed their adjuvant therapy a median (IQR) of 27.5 (30.8) months prior to the study.

**Table 1:** Demographics and clinical characteristics at study enrolment, stratified by patient group

	Group 1	Group 2	Group 3
	(n = 89)	(n = 108)	(n = 102)
Patient characteristics			
Mean (SD) age (years)	55.0 (11.1)	57.7 (10.6)	55.3 (11.2)
Ethnicity^a^ (n, %)			
White British	75 (84.3%)	101 (93.5%)	83 (81.4%)
Other	10 (11.2%)	7 (6.5%)	15 (14.7%)
Education^b^			
GCSE (or equivalent) or higher (n, %)	66 (74.2%)	81 (75.0%)	81 (79.4%)
No formal qualifications	21 (23.6%)	26 (24.1%)	19 (19.6%)
≥ 1 clinically significant comorbidities^c^, (n, %)	11 (12.4%)	10 (9.3%)	15 (14.7%)
Disease characteristics			
Tumour stage (n, %)			
Stage I	20 (22.5%)	25 (23.1%)	0 (0.0%)
Stage II	54 (60.7%)	53 (49.1%)	0 (0.0%)
Stage III	14 (15.7%)	29 (26.9%)	0 (0.0%)
Stage IV	0 (0.0%)	0 (0.0%)	101 (99.0%)
Not recorded	1 (1.1%)	0 (0.0%)	1 (1.0%)
Hormone receptor-positive (n, %)			
Yes	64 (71.9%)	84 (77.8%)	74 (72.5%)
No	25 (28.1%)	24 (22.2%)	26 (25.5%)
Not known	0 (0.0%)	0 (0.0%)	2 (2.0%)
Median (IQR) time since early BC diagnosis (months)	9.0 (6.0)	45.0 (32.0) (n = 103)	79.5 (82.0) (n = 71)
Median (IQR) time since metastatic BC diagnosis (months)			30.0 (37.0)
Type of metastatic BC diagnosis (n, %)			
De novo	–	–	27 (26.5%)
Relapsed	–	–	75 (73.5%)
Sites of metastatic BC disease (n*,* %)			
Non-visceral	–	–	23 (22.5%)
Visceral involvement	–	–	77 (75.5%)
Visceral involvement unknown	–	–	2 (2.0%)
No CNS involvement	–	–	71 (69.6%)
CNS involvement	–	–	25 (24.5%)
CNS involvement unknown	–	–	6 (5.9%)

n, numbers shown where data were not available or not applicable for all patients

*GCSE* General Certificate of Secondary Education, *IQR* interquartile range, *SD* standard deviation, *CNS* central nervous system

^a^Other ethnicities observed included “Other White”, “African”, “Caribbean”, “other Black”, “Chinese”, “Indian”, “Other Asian, and “Other ethnic group”; n = 4 patients in Group 1 and n = 4 patients in Group 3 did not state their ethnic group

^b^n = 2 patients in Group 1, n = 1 patient in Group 2 and n = 2 patients in Group 3 did not state their education level

^c^Excludes tumour and metastases

### Employment status and work productivity

Patient-reported employment status significantly differed between groups (χ^2^(1) = 39.45, *p* < 0.001; Table 2). Significantly more patients in Group 3, and significantly fewer patients in Group 2 reported inability to work (post hoc *p* < 0.003 for each comparison, significant after Bonferroni correction) than expected under the assumption of independence. Conversely, significantly fewer patients in Group 3 (post hoc *p* < 0.001), and marginally more patients in Group 2 (post hoc *p* = 0.03, not significant after Bonferroni correction) reported being in employment. For patients in part-time employment, the number of part-time hours worked per week reported by patients differed significantly between groups (F(2, 50) = 5.64; *p* = 0.006); patients in Group 3 reported working significantly fewer hours compared to those in Group 2 (post hoc *p* = 0.002, significant after Bonferroni correction; Table 2).

**Table 2:** Employment status and number of part-time hours worked by patient group

	Group 1	Group 2	Group 3	Between-group differences
				*p* value^d^
Employment status (n, %)				
Employed (full-time, part-time and self-employed)	45 (50.6)	55 (50.9)*	28 (27.5)**	< 0.001
Not employed^a^	41 (46.1)	52 (48.1)	69 (67.6)	
Retired	22 (24.7)	39 (36.1)	33 (32.4)	
Unable to work	7 (7.9)	5 (4.6)**	27 (26.5)**	
Not stated^b^	12 (13.5)	8 (7.4)	9 (8.8)	
Unknown	3 (3.4)	1 (0.9)	5 (4.9)	
Part-time hours worked per week (mean, SD)^c^	19.45 (6.04) (n = 20)	22.94 (6.36) (n = 20)	15.15 (7.44) (n = 13)	0.006

n, numbers are shown where data were not available or not applicable for all patients. Italic values denote observed values are significantly different to those ‘expected’ under the assumption of independence in post hoc tests, at thresholds of *p < 0.05, **p < 0.003 and ***p < 0.001

^a^Includes patients who reported “being housewives”, “out of work and looking for work”, or “out of work but not currently looking for work”

^b^Includes seven patients who selected more than one option

^c^Responses from patients who worked part-time only

^d^P-values are χ^2^ *p* value for employment status and ANOVA *p* value for part-time hours worked per week

**Fig. 1:**
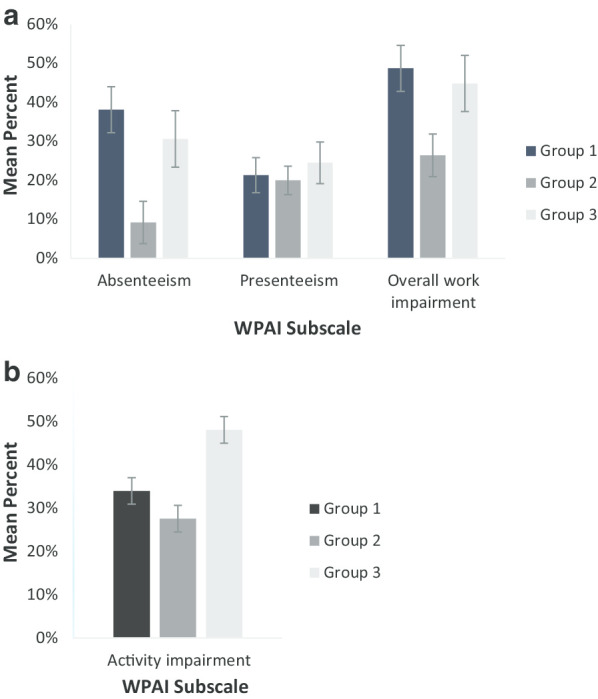
Impaired work and non-work productivity by patient group. (**a**) Impaired work activity reported by employed patients. (**b**) Impaired non-work activity reported by all patients. Error bars show the standard error of the mean (see Additional file 1: Table S3 for further details). Absenteeism corresponds to percentage of work time missed, presenteeism corresponds to percentage of impairment while working, and work productivity corresponds to overall work impairment due to health. *WPAI* work productivity and activity impairment

Groups differed significantly on the WPAI subscales of absenteeism (F(2, 71.218) = 6.23; *p* = 0.003), overall work impairment (*F*(2, 80.107) = 4.04; *p* = 0.021), and activity impairment (*F*(2, 277.666) = 13.81; *p* < 0.001), but not on presenteeism (*F*(2, 97) = 0.25; *p* = 0.781) (Fig. 1, Additional file 1: Table S3). In particular, employed patients in Group 2 reported a significantly lower proportion of absenteeism than those in Group 1 (post hoc *p* < 0.001), and a significantly lower proportion of overall work impairment compared to those in Group 1 (post hoc *p* = 0.015). Conversely, considering both employed and non-employed patients, patients in Group 3 reported a significantly higher proportion of activity impairment than those in Groups 1 (post hoc *p* = 0.004) and 2 (post hoc *p* < 0.001), which did not differ significantly from one another.

### Health-related quality of life

#### EQ-5D-5L

**Fig. 2:**
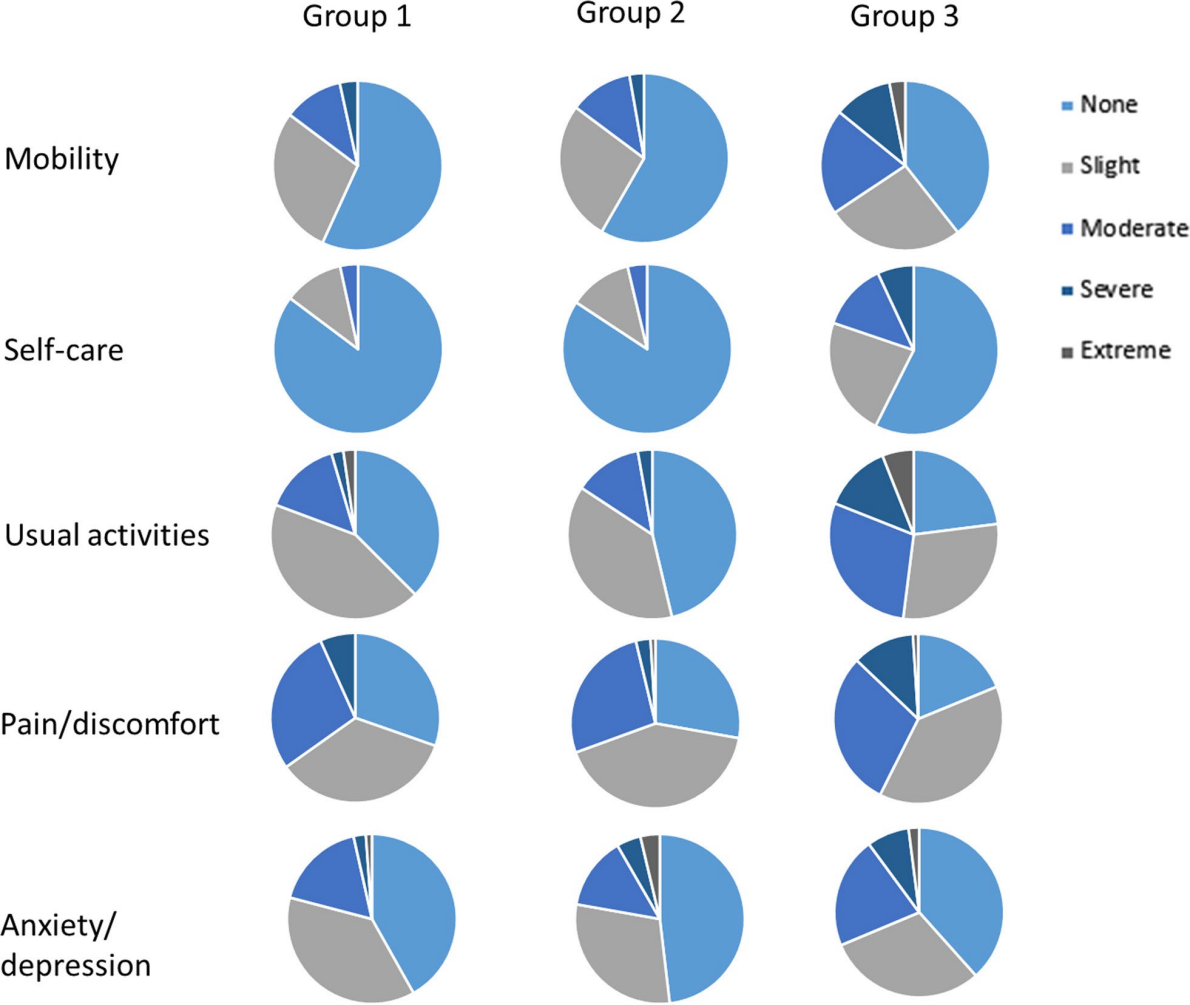
Proportion of patient responses by extent of problems reported in each EQ-5D-5L domain by patient group. See Additional file 1: Table S4 for further details

Health profiles, as assessed by questionnaire dimensions of the EQ-5D-5L, differed by groups. Ordinal regression analyses revealed that Group significantly predicted EQ-5D-5L ratings of mobility, self-care and usual activity (χ^2^(2) = 15.005, *p* = 0.001 for mobility scores, χ^2^(2) = 28.874, *p* < 0.001 for self-care scores, and (χ^2^(2) = 30.659, *p* < 0.001 for usual activity scores). Specifically, Groups 1 and 2 were more likely to report lower levels of impairment than Group 3, but not pain or discomfort (χ^2^(2) = 5.799, *p* = 0.055), nor anxiety and depression (χ^2^(2) = 2.809, *p* = 0.245) (Fig. 2, Additional file 1: Table S4). Generally, patients in Group 3 more commonly reported moderate or worse problems across the various EQ-5D-5L domains compared to patients in Groups 1 and 2, and particularly in the domains of self-care and usual activities.

Overall HRQoL, as assessed by EQ-5D-5L self-rated health status (assessed by VAS) and health utility (assessed by index values), also significantly differed across the patient groups (*Fisher* *p* < 0.001 for England tariff and VAS; Table 3 and Additional file 1: Table S5). The VAS scores reported by patients in Group 3 were significantly worse than those reported by patients in Group 2 (post hoc *p* < 0.001), and were marginally worse than those reported by patients in Group 1 (post hoc *p* = 0.059); while Group 1 and Group 2 did not differ significantly from one another. Patients in Group 3 reported significantly poorer health utility than those in Group 1 (England tariff post hoc *p* = 0.002) and Group 2 (England tariff post hoc *p* < 0.001); while Group 1 and Group 2 did not differ significantly from one another.

**Table 3:** Self-rated health status and health utility as measured by the EQ-5D-5L by patient group

	Group 1	Group 2	Group 3	Between-group differences
				Fisher *p* value
Utility weighted by the England tariff	0.809 (0.170) (n = 86)	0.818 (0.181) (n = 108)	0.695 (0.262) (n = 97)	< 0.001
Visual analogue scale	72.74 (18.39) (n = 89)	77.01 (17.53) (n = 108)	65.82 (22.86) (n = 99)	< 0.001

Higher scores reflect higher levels of HRQoL. n, numbers shown where data were not available for all patients. See Additional file 1: Table S5 for index values estimated using the UK crosswalk tariff

#### FACT-B

Groups differed significantly on the physical wellbeing (F(2,268.753) = 10.464, *p* < 0.001), social wellbeing (F(2,277.928) = 6.784, *p* = 0.001), emotional wellbeing (F(2,278.229) = 8.787, *p* < 0.001) and functional wellbeing (F(2,273.811) = 10.464, *p* < 0.001) subscales, as well as all of the summary scores (FACT-G: F(2,266.591) = 11.327, *p* < 0.001; FACT-B: F(2,289) = 8.172, *p* < 0.001; trial outcomes index: F(2,293) = 7.434, *p* = 0.001); although not significantly on the breast cancer-specific subscale (*p* = 0.602; Table 4). In particular, Group 2 reported significantly better physical wellbeing than Group 1 [post hoc *p* = 0.031 (not significant after Bonferroni correction)] and Group 3 (post hoc *p* < 0.001); which also represented a clinically significant difference (exceeding the established minimal important difference [MID] of 2 points). Group 2 also reported clinically and statistically significantly better functional wellbeing than Group 1 (post hoc *p* = 0.044) and Group 3 (post hoc *p* < 0.001), which differed marginally from one another (post hoc *p* = 0.060). Group 1 reported clinically and statistically significantly better social wellbeing than Group 3 (post hoc *p* = 0.001). Group 3 reported clinically and statistically significantly poorer emotional wellbeing than Group 1 (post hoc *p* = 0.001) and Group 2 (post hoc *p* = 0.004), which did not differ statistically from one another. Group 3 scored clinically and statistically significantly worse on the FACT-G, FACT-B summary scores than Group 1 (post hoc *p* = 0.003 and post hoc *p* = 0.004, respectively) and Group 2 (both post hoc *p* < 0.001), which did not differ statistically from one another. Group 3 also scored clinically and statistically significantly worse on the trial outcomes index than Group 2 (post hoc *p* < 0.001), although only marginally different than Group 1 (post hoc *p* = 0.068; post hoc *p* = 0.067 for Group 1 vs Group 2).

**Table 4:** Health-related quality of life as measured by the FACT-B scores by patient group

	Group 1	Group 2	Group 3	Between-group differences
				Fisher *p* value
FACT-B subscales (mean, SD)				
Physical wellbeing	20.57 (5.93) (n = 86)	22.63 (5.08) (n = 107)	18.81 (6.89) (n = 101)	< 0.001
Social wellbeing	23.62 (4.88) (n = 86)	22.26 (5.23) (n = 108)	20.65 (6.34) (n = 100)	0.001
Emotional wellbeing	18.13 (4.87) (n = 87)	17.68 (4.87) (n = 107)	15.19 (5.99) (n = 100)	< 0.001
Functional wellbeing	18.69 (6.32) (n = 88)	20.76 (5.52) (n = 108)	16.43 (7.30) (n = 101)	< 0.001
Breast cancer-specific	22.01 (7.00) (n = 86)	22.38 (7.68) (n = 108)	21.37 (6.96) (n = 101)	0.602
FACT-B summary scores (mean, SD)				
FACT-G^a^	80.89 (17.69) (n = 85)	83.19 (16.80) (n = 106)	71.07 (22.42) (n = 99)	< 0.001
FACT-B total	102.93 (23.41) (n = 85)	105.58 (23.00) (n = 106)	92.24 (27.38) (n = 99)	< 0.001
Trial outcome index^b^	61.19 (16.95) (n = 86)	65.74 (16.05) (n = 107)	56.61 (18.14) (n = 101)	0.001

Higher scores reflect higher levels of HRQoL. n, numbers shown where data were not available for all patients

^a^FACT-G: Functional Assessment of Cancer Therapy-General; constitutes the non-tumour specific core of the FACT-B subscale [18]

^b^Trial outcome index calculated from the sum of the physical wellbeing, functional wellbeing, and additional concerns subscales of the FACT-B [18]

### Relationship between productivity and health-related quality of life

Higher activity impairment and overall work impairment were significantly associated with poorer HRQoL, as assessed by EQ-5D-5L VAS, and FACT-B total and FACT-G scores (all *p* < 0.001), and the strongest associations were observed for activity impairment (Additional file 1: Table S6).

The correlations between FACT-B domains and absenteeism and presenteeism are presented in Additional file 1: Table S7. Poorer physical and functional wellbeing and breast cancer-specific symptoms were significantly associated with higher impairment in work productivity as measured by levels of absenteeism and presenteeism (Pearson’s correlations *p* < 0.001); while social wellbeing was not observed to be associated with either domain of work productivity. In a multiple linear regression model, physical wellbeing, functional wellbeing and breast cancer specific symptoms significantly predicted absenteeism (*p* < 0.001), and collectively explained 24% of the variance in absenteeism (Additional file 1: Table S8). Of these predictors, only functional wellbeing was a significant independent predictor. Physical wellbeing, emotional wellbeing, functional wellbeing and breast cancer specific symptoms significantly predicted presenteeism (*p* < 0.001), and collectively explained 53% of the variance in presenteeism. Of these predictors, both physical and functional wellbeing were significant independent predictors.

## Discussion

Several published studies have evaluated the impact of breast cancer on HRQoL in patients with HER2-positive metastatic BC [21], or patients with BC at different stages of disease [22], and compared stages of BC in terms of loss of work productivity [12]. However, this is, to our knowledge, the first study conducted in patients with HER2 BC describing both HRQoL and productivity in relation to different stages of disease and treatment. In addition, although studies have been published in Asian [22], North-American [2] and some European populations [12] on these aspects, evidence remains limited for the UK.

### Breast cancer in relation to employment and work productivity

Metastatic disease was found to impact employment status, with approximately one quarter of patients with metastatic disease (Group 3) reporting being unable to work, compared with fewer than one in ten patients with early BC (Groups 1 and 2). When in part-time employment, patients in Group 3 also worked fewer hours than patients in Groups 1 and 2. In a recent meta-analysis, breast cancer survivors remained at a significantly higher risk of unemployment compared to the general population, which was not the case for other cancers [23]. This may be because the other cancers (blood cancers and testicular cancers) are more common in younger patients, and that perhaps patients with BC were opting for early retirement. However, it is notable that despite lower rates of employment in patients with metastatic BC, of the patients in employment, there were no significant differences in terms of presenteeism between the three groups, suggesting that patients who were able to work, even part-time, were able to be productive. Patients with metastatic BC had similar levels of overall work impairment compared to patients with early BC on treatment. The main impact on productivity was from increased absenteeism in both groups, suggesting that loss of working days due to treatment was an important reason for impaired productivity and would decrease once the patients entered remission. Two studies conducted in North America have reported on loss of working days in women with BC: in the US an average of 22 days were missed from work, and 40 days in patients at more advanced stage of disease [24]; and in Canada patients took almost 6 months off work on average [25]. Further studies would be warranted to better understand absenteeism in patients with breast cancer and how the impact of treatment and stage of disease on work attendance and productivity may be minimised.

Levels of overall work impairment and activity impairment were below 50% in all groups in our study. This is relatively lower than previously reported in Sweden and the Netherlands where levels of work-productivity impairment among women currently receiving treatment for BC were 72% and 69%, respectively, and levels of activity impairment were 62% and 55%, respectively [12]. However, our estimates are similar to those of a US study in patients with advanced BC where activity impairment was 30% and work-productivity impairment ranged between 20 and 40% for the different WPAI scores in employed patients [2]. This emphasises the importance of determining country-specific measurements for estimating the impact of BC on productivity. Patients in remission in our study were significantly less impaired than patients on treatment for subscale scores of work productivity (absenteeism, overall work impairment and activity impairment, not significantly for presenteeism), consistent with the previous study from Sweden and the Netherlands [12]. However, even in patients in remission, absence due to disease and impairment whilst at work remained at levels impacting productivity, emphasising the need for long-term support for women going back to work after treatment for BC.

### Breast cancer in relation to health-related quality of life

Patients with early BC experienced similar levels of HRQoL, whether they were in remission or on treatment, and significantly higher HRQoL than patients with metastatic BC. Similarly, in a meta-analysis in Asian women, patients with BC with comorbidities and those treated with chemotherapy had poorer HRQoL compared to women at earlier stages of disease [22]. In a study of US women with HER2-positive metastatic BC, levels of HRQoL increased over time, with women living longer with the disease experiencing an improvement in HRQoL compared to women diagnosed more recently [21]. Levels of HRQoL, as indicated by the EQ-5D-5L and FACT-B summary scores, were similar to that previously reported in other countries, confirming that patients with BC have relatively high levels of HRQoL [2, 13, 26]. In a previous study in Sweden and the Netherlands mean utility scores for stable and progressive diseases were 0.81 and 0.61, respectively [12], similar to utility scores in our sample as estimated with the England tariff. However, it is notable that the FACT-B breast cancer-specific subscale scores were similar between the three groups, suggesting this subscale is not sensitive to differences in breast cancer stage or treatment, consistent with results of the original validation paper that demonstrated no association of this subscale with extent of disease [27].

In our study, disease stage did not explain the level of anxiety and depression, which was not a concern for approximately 40–50% of patients. This is in line with a study demonstrating a majority of women with advanced disease remaining below clinical thresholds for depression [14], and another study reporting high levels of emotional wellbeing in patients in long-term remission [13]. In a meta-analysis, symptoms of depression were elevated in patients in remission 1-year after treatment, and decreased over the ensuing years; and anxiety was not raised in any year post-treatment [28]. In contrast, in our study, anxiety and depression in patients with early BC were similar in those on treatment (diagnosed on average 6 months prior to study enrolment) and in patients in remission (diagnosed on average 2.7 years prior to study enrolment). However, it should be acknowledged that poorer emotional wellbeing, as assessed by FACT-B, was observed in patients with metastatic BC compared with those with early BC. While our study was not adequately powered to further investigate the impact of time in remission on anxiety and depression, our results suggest that, although treatment for BC impacts on various aspects of daily life, including emotional wellbeing and productivity, the majority of women at all stages of BC maintain good mental health in terms of anxiety and depression, even in the presence of metastatic disease.

### Relationship between health-related quality of life and work productivity in patients with breast cancer

Poorer HRQoL was associated with greater work and non-work impairment: physical and functional wellbeing, along with BC specific symptoms, were correlated with absenteeism and presenteeism, and emotional wellbeing was associated with presenteeism only. This is in line with the findings of a previous study, which showed that overall quality of life in patients with BC in remission was associated with economic aspects such as work productivity, quality of work, absenteeism, and change in spouse/partner’s income [29]. A recent study in patients with HER2-positive metastatic BC observed a temporal adjustment to the impact of disease, with patients having lived longer with the disease experiencing higher levels of HRQoL and also reporting lower productivity impairment than those recently diagnosed [21]. Taken together, this evidence suggests that maintaining high levels of HRQoL in patients with BC may have a positive economic impact, and conversely limiting the economic burden in patients with BC may translate into improved HRQoL, at all stages of disease and treatment.

### Strengths and limitations

Our study has several strengths. Characteristics of patients in our study were similar to UK patients with HER2-positive BC as reported nationally with regard to age, ethnicity, and educational status [30, 31]. A quarter of patients in Group 3 were diagnosed de novo with metastatic BC. This is within the range reported in other studies, including a study in the Netherlands which observed 19% of patients diagnosed with de novo metastatic BC [32], and a study in US patients which reported 33% with de novo metastatic BC [33].

This study is subject to limitations. Selection bias may have been introduced by including consenting patients only, as they may have differed from patients refusing consent. Two thirds of the patients included in our study were not in paid employment, the majority of which were retired, and therefore some analyses in employed patients will have been conducted in small samples. This was a cross-sectional study and therefore the temporal relationship between disease/treatment stage and impact on productivity and HRQoL could not be assessed. In particular, it was not possible to conclude whether worse HRQoL and productivity for patients with metastatic BC (three quarters of which were not de novo metastatic and had progressed from early BC) compared to patients with early BC was due to differences between individuals rather than to worsening of patient’s condition with disease progression. Similarly, the better HRQoL in patients with early BC in remission compared to those still on treatment could be attributable in part to sample variation rather than temporal improvement. Furthermore, we did not evaluate the relationship between the time since diagnosis and HRQoL. In the population of patients in the study with metastatic disease, approximately one quarter had central nervous system (CNS) disease. This is consistent with the reported incidence of brain metastases in HER-2 positive breast cancer [34]. The presence of CNS disease would be expected to have a disproportionate impact on HRQoL and work productivity compared to extracranial disease sites. Furthermore, the study relied on the completeness and quality of medical records and the patients’ answers to questionnaires.

Whilst a longitudinal study design could identify temporal changes in HRQoL and work productivity throughout the course of disease, significant loss to follow-up would be expected during a prolonged period of observation, which would limit the interpretation of results, supporting the cross-sectional approach.

It should be noted that analyses reported were not corrected for potential confounders because groups did not differ on demographic variables or comorbidities and other covariate variables were considered to be either intrinsically linked to group selection criteria (e.g. treatment or disease history), or to the variables being assessed (employment status is somewhat dependent on HRQoL). However, we cannot exclude that other factors not evaluated in the present study (e.g. time since diagnosis) may have influenced the study results. The results of this study should be interpreted in light of these limitations.

## Conclusion

The results of the present study in UK patients with HER2-positive BC are broadly consistent with results of previous studies in Asian, North American and European patients with BC. In particular, metastatic disease and treatment of HER2-positive BC were shown to adversely impact on both work productivity and HRQoL. The results of this study support the idea that being able to delay or prevent the metastatic recurrence of BC, for example by extending the time patients are in remission or at an early stage of BC, has wider benefits in terms of patient productivity and HRQoL.

## Supplementary information

**Additional file 1**. Supplementary information and analyses.

## Data Availability

The data that support the findings of this study are available from Roche Products Ltd but restrictions apply to the availability of these data, which were used under license for the current study, and so are not publicly available. Data are however available from the authors upon reasonable request and with permission of Roche Products Ltd.

## Abbreviations

ANOVA: Analysis of variance
BC: Breast cancer
CNS: Central nervous system
EQ-5D-5L: EuroQoL-5 dimension-5 level
FACT-B: Functional Assessment of Cancer Therapy-Breast Cancer
FACT-G: Functional Assessment of Cancer Therapy-General
GCSE: General Certificate of Secondary Education
HER2: Human epidermal growth factor 2
HRQoL: Health-related quality of life
IQR: Interquartile range
SD: Standard deviation
VAS: Visual analogue scale
WPAI: Work productivity and activity impairment
