# Supplementary material for: Health-related quality of life and work productivity in UK patients with HER2-positive breast cancer: a cross-sectional study evaluating the relationships between disease and treatment stage
Source: Health Qual Life Outcomes. 2020 Nov 2;18:353. doi: 10.1186/s12955-020-01603-w (PMC7607622; doi:10.1186/s12955-020-01603-w)
Supplement: Supplementary file 1 — Additional file 1. Supplementary information and analyses. [file 12955_2020_1603_MOESM1_ESM.docx]

**Additional file Tables**

Table S1. Patient comorbidities by subgroup

|  | Group 1  *n*=89 | Group 2  *n*=108 | Group 3  *n*=102 | Between-group differences (*p*) |
| --- | --- | --- | --- | --- |
| Clinically significant comorbidities (*n*, %) | | | | |
| One or more (excluding tumour or metastases) | 11 (12.4%) | 10 (9.3%) | 15 (14.7%) | 0.477 |
| Peripheral arterial disease (includes aortic aneurysm ≥6 cm) | 2 (2.2%) | 0 (0.0%) | 1 (1.0%) | 0.289 |
| Cerebrovascular disease (cerebrovascular accident with mild or no residual impairment, or transient ischaemic attacks) | 0 (0.0%) | 0 (0.0%) | 1 (1.0%) | 0.379 |
| Chronic pulmonary disease | 3 (3.4%) | 2 (1.9%) | 1 (1.0%) | 0.496 |
| Connective tissue disease | 1 (1.1%) | 3 (2.8%) | 0 (0.0%) | 0.211 |
| Peptic ulcer disease | 1 (1.1%) | 0 (0.0%) | 0 (0.0%) | 0.306 |
| Moderate or severe renal disease | 1 (1.1%) | 0 (0.0%) | 3 (2.9%) | 0.175 |
| Leukaemia (acute or chronic) | 1 (1.1%) | 1 (0.9%) | 0 (0.0%) | 0.585 |
| Lymphoma | 1 (1.1%) | 0 (0.0%) | 1 (1.0%) | 0.562 |
| Tumour without metastasis (exclude if >5 years from diagnosis) | 29 (32.6%) | 10 (9.3%) | 0 (0.0%) | - |
| Metastatic solid tumour | 0 (0.0%) | 0 (0.0%) | 56 (54.9%) | - |
| Mild liver disease (without portal hypertension, includes chronic hepatitis) | 0 (0.0%) | 1 (0.9%) | 3 (2.9%) | 0.189 |
| Moderate or severe liver disease | 0 (0.0%) | 0 (0.0%) | 1 (1.0%) | 0.379 |
| Diabetes without end-organ damage (excludes diet-controlled alone) | 3 (3.4%) | 4 (3.7%) | 6 (5.9%) | 0.641 |
| Diabetes with end-organ damage (retinopathy, neuropathy, nephropathy or brittle diabetes) | 0 (0.0%) | 0 (0.0%) | 2 (2.0%) | 0.143 |

Note: where appropriate, variables were compared using a chi-square test. Between-group differences were not significant for any of the comorbidities examined.

**Table S2. Current treatment status by patient group**

|  | Group 1  *n*=89 | Group 2  *n*=108 | Group 3  *n*=102 |
| --- | --- | --- | --- |
| Current line of metastatic therapy (*n*, %) | | | |
| First-line metastatic | 0 (0.0%) | 0 (0.0%) | 55 (53.9%) |
| Second-line metastatic | 0 (0.0%) | 0 (0.0%) | 22 (21.6%) |
| Third-line metastatic | 0 (0.0%) | 0 (0.0%) | 10 (9.8%) |
| Fourth-line metastatic | 0 (0.0%) | 0 (0.0%) | 4 (3.9%) |
| Later than fourth-line metastatic | 0 (0.0%) | 0 (0.0%) | 11 (10.8%) |
| Currently receiving chemotherapy? | | | |
| Yes | 27 (30.3%) | 0 (0.0%) | 29 (28.4%) |
| No (not applicable) | 62 (69.7%) | 108 (100.0%) | 73 (71.6%) |
| Currently receiving HER2 targeted therapy? | | | |
| Yes | 88 (98.9%) | 0 (0.0%) | 96 (94.1%) |
| With chemotherapy | 27 (30.3%) | — | 24 (24.5%) |
| Without chemotherapy | 61 (68.5%) | — | 72 (69.6%) |
| No (not applicable) | 1 (1.1%) | 108 (100.0%) | 6 (5.9%) |
| Currently receiving hormone therapy? | | | |
| Yes | 40 (44.9%) | 66 (61.1%) | 17 (16.7%) |
| No (not applicable) | 49 (55.1%) | 42 (38.9%) | 85 (83.3%) |
| Currently receiving investigational treatments? | | | |
| Not applicable | 89 (100.0%) | 108 (100.0%) | 100 (98.0%) |
| Investigational immunotherapy | 0 | 0 | 2 (2.0%) |
| Duration of current therapy (months) | | | |
| Median (IQR) | 5.0 (6.0) | 26.0 (30.0) (*n*=51) | 12.0 (22.0) (*n*=101) |
| Months since adjuvant therapy completed | | | |
| Median (IQR) | — | 27.5 (30.8) | — |

*n*, numbers shown where data were not available for all patients.

IQR: interquartile range.

**Table S3: Work Productivity and Activity Impairment Subscale scores by patient subgroup**

| WPAI Subscale Scores (Mean, SD) | Group 1 | Group 2 | Group 3 | Between-group differences  Fisher p-value |  |
| --- | --- | --- | --- | --- | --- |
|  |  |  |  |  |  |
| Percentage of work time missed due to ill  health (absenteeism)^a^ | | 38.1% (43.8%) (*n*=39) | **9.2% (23.5%)^***^** (*n*=46) | 30.6% (43.9%) (*n*=26) | 0.003 |
| Percentage of impairment while working  (presenteeism)^a^ | | 21.3% (24.2%) (*n*=31) | 20.0% (22.7%) (*n*=47) | 24.5% (30.5%) (*n*=22) | 0.781 |
| Overall work impairment due to health (work  productivity) | | 48.7% (39.7%) (*n*=39) | **26.4% (30.5%)^***^** (*n*=45) | 44.8% (41.7%) (*n*=26) | 0.021 |
| Activity impairment^b^ | | 34.0% (28.0%) (*n*=86) | **27.6% (26.1%)^***^** (*n*=107) | **48.1% (31.4%)^***^** (*n*=100) | <0.001 |

^a^Applicable to employed patients only. ^b^Applicable to all patients. *n*, numbers shown where data were not available for all patients.

WPAI:Work Productivity and Activity Impairment.

**Table S4: EQ-5D-5L patient responses by subgroup**

| EQ-5D domains | Group 1 | Group 2 | Group 3 |
| --- | --- | --- | --- |
|  |  |  |  |
| Mobility | *n*=88 | *n*=108 | *n*=99 |
| No problems | 50 (56.8%) | 63 (58.3%) | 39 (39.4%) |
| Slight problems | 25 (28.4%) | 29 (26.9%) | 26 (26.3%) |
| Moderate problems | 10 (11.4%) | 13 (12.0%) | 20 (20.2%) |
| Severe problems | 3 (3.4%) | 3 (2.8%) | 11 (11.1%) |
| Extreme problems | 0 (0.0%) | 0 (0.0%) | 3 (3.0%) |
| Self-care | *n*=88 | *n*=108 | *n*=101 |
| No problems | 75 (85.2%) | 91 (84.3%) | 58 (57.4%) |
| Slight problems | 10 (11.4%) | 13 (12.0%) | 23 (22.8%) |
| Moderate problems | 3 (3.4%) | 4 (3.7%) | 13 (12.9%) |
| Severe problems | 0 (0.0%) | 0 (0.0%) | 7 (6.9%) |
| Extreme problems | 0 (0.0%) | 0 (0.0%) | 0 (0.0%) |
| Usual activities | *n*=88 | *n*=108 | *n*=100 |
| No problems | 33 (37.5%) | 50 (46.3%) | 23 (23.0%) |
| Slight problems | 38 (43.2%) | 41 (38.0%) | 29 (29.0%) |
| Moderate problems | 13 (14.8%) | 14 (13.0%) | 29 (29.0%) |
| Severe problems | 2 (2.3%) | 3 (2.8%) | 13 (13.0%) |
| Extreme problems | 2 (2.3%) | 0 (0.0%) | 6 (6.0%) |
| Pain or discomfort | *n*=89 | *n*=108 | *n*=101 |
| No problems | 27 (30.3%) | 30 (27.8%) | 19 (18.8%) |
| Slight problems | 31 (34.8%) | 45 (41.7%) | 39 (38.6%) |
| Moderate problems | 25 (28.1%) | 29 (26.9%) | 30 (29.7%) |
| Severe problems | 6 (6.7%) | 3 (2.8%) | 12 (11.9%) |
| Extreme problems | 0 (0.0%) | 1 (0.9%) | 1 (1.0%) |
| Anxiety or depression | *n*=86 | *n*=108 | *n*=99 |
| No problems | 36 (41.9%) | 52 (48.1%) | 38 (38.4%) |
| Slight problems | 32 (37.2%) | 32 (29.6%) | 30 (30.3%) |
| Moderate problems | 15 (17.4%) | 15 (13.9%) | 21 (21.2%) |
| Severe problems | 2 (2.3%) | 5 (4.6%) | 8 (8.1%) |
| Extreme problems | 1 (1.2%) | 4 (3.7%) | 2 (2.0%) |

**Table S5:** Health utility as measured by the EQ-5D-5L summary scores by patient group

|  | Group 1 | Group 2 | Group 3 | Between-group differences |
| --- | --- | --- | --- | --- |
|  |  |  |  | *Fisher p-value* |
| Utility weighted by the UK crosswalk tariff | 0.728 (0.197)  (*n*=86) | 0.732 (0.216)  (*n*=108) | 0.603 (0.271)  (*n*=97) | <0.001 |

**Table S6. Relationships (Pearson’s *r*) between health-related quality of life and productivity impairment**

|  | **Overall work impairment**  ***n*=109** | **Activity impairment** |
| --- | --- | --- |
| EQ-5D VAS | –0.407 (*p*<0.001) | -0.671 (n=291) (*p*<0.001) |
| FACT-B total | –0.577 (*p*<0.001) | –0.679 (*n*=287) (*p*<0.001) |
| FACT-G | –0.533 (*p*<0.001) | –0.692 (*n*=287) (*p*<0.001) |

*n*, numbers shown where data were not available for all patients.

FACT-B: Functional Assessment of Cancer Therapy-Breast; FACT-G: Functional Assessment of Cancer Therapy–General.

**Table S7. Pearson’s correlations between FACT-B domains and work-related productivity impairment**

|  | **Absenteeism**  ***n*=111** | **Presenteeism**  ***n*=100** |
| --- | --- | --- |
| Physical wellbeing | –0.335 (*p*<0.001) | –0.660 (*p*<0.001) |
| Social wellbeing | 0.007 *n.s.* | –0.170 *n.s.* |
| Emotional wellbeing | –0.168 (*n*=110) *n.s.* | –0.522 (*n*=99) (*p*<0.001) |
| Functional wellbeing | –0.470 (*p*<0.001) | –0.589 (*p*<0.001) |
| Breast cancer specific symptoms | –0.365 (*p*<0.001) | –0.513 (*p*<0.001) |

Includes employed patients only. *n*, numbers shown where data were not available for all patients. n.s.: not significant.

**Table S8: Regression models between FACT-B domains and work-related productivity impairment**

|  | **Absenteeism**  ***R*^2^=0.24 (*p*<0.001)** | **Presenteeism**  ***R*^2^=0.53 (*p*<0.001)** |
| --- | --- | --- |
| **Predictor** | ***β*** | ***β*** |
| Physical wellbeing | –0.065 *n.s.* | –0.438 (*p*<0.001) |
| Functional wellbeing | –0.358 (*p*<0.01) | *.* –0.227 (*p*<0.05) |
| Breast cancer-specific symptoms | –0.138 *n.s.* | –0.124 *n.s.* |
| Emotional wellbeing | — | –0.068 *n.s* |

The model was run selecting employed patients only. n.s.: not significant.
